# Supplementary material for: A fault-intrusion-tolerant system and deadline-aware algorithm for scheduling scientific workflow in the cloud
Source: PeerJ Comput Sci. 2021 Nov 2;7:e747. doi: 10.7717/peerj-cs.747 (PMC8576554; doi:10.7717/peerj-cs.747)
Supplement: Supplemental Information 6 [file peerj-cs-07-747-s006.zip › Myproject1.1/docs/org/cloudbus/cloudsim/provisioners/class-use/PeProvisioner.html]

Uses of Class org.cloudbus.cloudsim.provisioners.PeProvisioner


JavaScript is disabled on your browser.


- Overview
- Package
- Class
- Use
- Tree
- Deprecated
- Index
- Help

- Prev
- Next

- Frames
- No Frames

- All Classes

## Uses of Class org.cloudbus.cloudsim.provisioners.PeProvisioner

- Packages that use PeProvisioner

  | Package | Description |
  | --- | --- |
  |  |  |
  | --- | --- |
  | org.cloudbus.cloudsim |  |
  | org.cloudbus.cloudsim.provisioners |  |
- - ### Uses of PeProvisioner in org.cloudbus.cloudsim

    Methods in org.cloudbus.cloudsim that return PeProvisioner

    | Modifier and Type | Method and Description |
    | --- | --- |
    |  |  |
    | --- | --- |
    | `PeProvisioner` | Pe.`getPeProvisioner()` Gets the Pe provisioner. |

    Methods in org.cloudbus.cloudsim with parameters of type PeProvisioner

    | Modifier and Type | Method and Description |
    | --- | --- |
    |  |  |
    | --- | --- |
    | `protected void` | Pe.`setPeProvisioner(PeProvisioner peProvisioner)` Sets the pe provisioner. |

    Constructors in org.cloudbus.cloudsim with parameters of type PeProvisioner

    Constructor and Description

    `Pe(int id,
    PeProvisioner peProvisioner)`

    Allocates a new Pe object.
  - ### Uses of PeProvisioner in org.cloudbus.cloudsim.provisioners

    Subclasses of PeProvisioner in org.cloudbus.cloudsim.provisioners

    | Modifier and Type | Class and Description |
    | --- | --- |
    |  |  |
    | --- | --- |
    | `class` | `PeProvisionerSimple` The Class PeProvisionerSimple. |

- Overview
- Package
- Class
- Use
- Tree
- Deprecated
- Index
- Help

- Prev
- Next

- Frames
- No Frames

- All Classes
